# Cutting-edge choices: comparative outcomes of laparoscopic vs robotic cholecystectomy—a systematic review

**DOI:** 10.1007/s13304-026-02671-1

**Published:** 2026-06-01

**Authors:** En Qing Lim, Zeeshan Razzaq

**Affiliations:** Department of Surgery, Cork University Hospital, Cork, Republic of Ireland

**Keywords:** Laparoscopic cholecystectomy, Robotic-assisted cholecystectomy, Gallbladder surgery, Minimally invasive surgery, Surgical efficacy

## Abstract

Minimally invasive techniques, particularly laparoscopic cholecystectomy (LC) and robotic-assisted cholecystectomy (RAC), are now the mainstays in the surgical management of gallbladder diseases. Both techniques are widely used, but there is a paucity of comparative studies with regards to surgical efficacy, cost, and patient recovery time. This meta-analysis aims to compare their key differences to improve clinical practice and outcomes. A PRISMA-compliant systematic review was conducted of peer-reviewed comparative studies evaluating LC versus RAC for benign gallbladder disease. Outcomes included conversion to open surgery, operative time, complication rates, postoperative length of stay, postoperative pain, and cost-effectiveness. Random-effects meta-analysis was performed where appropriate.Six studies were included. RAC was associated with significantly lower odds of conversion to open surgery than LC (OR 0.10, 95% CI 0.01–0.94; *p* = 0.04), although this finding was based on sparse retrospective data. RAC was associated with longer operative time in the available comparative data. No significant difference was found in postoperative length of stay (MD 0.38 days, 95% CI −0.06 to 0.82; *p* = 0.09) or overall complication rates (OR 0.57, 95% CI 0.04–7.79; *p* = 0.67). Pain outcomes were reported using non-standardized measures, limiting firm interpretation. Most studies suggested RAC was more costly than LC, although findings varied by institutional setting and costing method. RAC may reduce conversion to open surgery in selected settings, but broader clinical benefit over LC remains uncertain. Overall, LC remains the more established and economically justifiable approach for most patients undergoing cholecystectomy for benign gallbladder disease, while further high-quality prospective studies are needed to clarify the role of RAC.

## Introduction

Minimally invasive surgical procedures have revolutionized the treatment of gallbladder diseases by introducing techniques that reduce patients’ morbidity and additionally improve recovery times. In this regard, laparoscopic cholecystectomy (LC) has been long established as a standard of care due to its cost-effectiveness, safety, and effectiveness in non-complicated cases [1, 2]. However, advancements in robotics have led to the introduction of robotic-assisted cholecystectomy (RAC), which provides more dexterity, better visualization, and higher precision, mostly in complex cases [3, 4].

Benign gallbladder diseases including biliary colic, acute cholecystitis, and biliary dyskinesia, are commonly encountered medical conditions that necessitate surgical treatment. While LC remains a preferred technique for uncomplicated cases, RAC has been increasingly used in scenarios where technical challenges require superior surgical control in order to minimize conversion to open surgery [5, 6]. The rise of robotic-assisted surgery has brought considerable attention, with proponents highlighting its usefulness in managing complex surgical pathologies and opponents pointing out its longer surgical times and higher costs [1, 3].

A comparative analysis of LC and RAC reveals both shared benefits and distinct limitations. RAC is associated with lower conversion rates, faster postoperative recovery, and greater technical precision in the treatment of complex cases [5, 6]. However, the longer operative time entailed by RAC, which is generally about 20 min longer than LC, and its significantly higher costs have hindered its more widespread adoption [2, 4]. Moreover, while robotic-assisted surgery is promising to improve surgical outcomes, its ecological footprint and feasibility in low-resource settings need much more critical scrutiny [4, 7].

The aim of this study is to compare key differences between LC and RAC to improve clinical practice and outcomes.

## Methods

This systematic review was performed in accordance with the Preferred Reporting Items for Systematic Reviews and Meta- Analysis (PRISMA) statement [8].

### Literature search

We conducted a systematic search of three databases: PubMed, Embase and Google Scholar, using the following search terms as MeSH terms or in all fields:

“Laparoscopic cholecystectomy” OR “LC”.

AND

“Robotic-assisted cholecystectomy” OR “RAC”.

AND

“Gallbladder diseases’’.

Studies from inception of each database up until November 2024, of which only those available in English and full text were considered for inclusion.

### Inclusion criteria

Studies involving adult patients (≥ 18 years) undergoing cholecystectomy for benign gallbladder diseases such as symptomatic cholelithiasis, cholecystitis or biliary dyskinesia were included in this systematic review.

In terms of study design, randomized controlled trials and comparative observational studies, including prospective or retrospective cohort studies and case–control studies, were eligible for inclusion, provided they directly compared laparoscopic and robotic-assisted cholecystectomy in adult patients with benign gallbladder disease.

### Exclusion criteria

Non-comparative studies, including case reports, case series, review articles, conference abstracts without full text, and studies not directly comparing laparoscopic with robotic-assisted cholecystectomy, were excluded.

### Selection process

Two review authors worked independently to screen the titles and abstracts of articles obtained from the three databases (PubMed, Embase and Google Scholar), following which irrelevant and duplicate studies were removed.

The remaining articles were then screened against the inclusion and exclusion criteria, with the two review authors once again working independently in the process.

A final list of articles was produced, and any discrepancies included were resolved through discussion between two authors, and the third review author was consulted if a discussion between the two review authors did not result in an agreement.

### Data collection

Two review authors worked independently to collect data from eligible studies, and disagreements were resolved with discussion and involvement of the third author if needed. The following data were collected from each study (Table 1):

- Author, year of publication, study design, country of origin
- Characteristics of patients: age, sex, comorbidities
- Sample size of study
- Type of intervention: Laparoscopic or Robotic-assisted cholecystectomy (The included studies evaluated different robotic techniques, including single-site robotic cholecystectomy and broader robotic-assisted approaches.)
- Operative time
- Conversion rates
- Post-op length of hospital stay
- Complications relating to the procedure
- Postoperative Pain
- Cost-Effectiveness

**Table 1:** Characteristics of included studies. Continuous variables are presented as mean ± SD or median (range)

Author, year of publication	Country(s) of origin	Type of study	Sample size, *n*	Age (LC: RAC, years)	Male, n (%)	Type of Intervention
Pokala et al., [2]	USA	Retrospective Cohort	Total: 91,849 LC: 89,878 RAC: 1,971	LC: 18–30 (17.9%), 31–50 (35.1%), 51–64 (23.4%), > 65 (23.6%) RAC: 18–30 (10.9%), 31–50 (35.5%), 51–64 (26.9%), > 65 (26.7%)	LC: 30,194/ 89,878 (33.6%); RAC: 660/ 1971 (33.5%)	Robotic-Assisted, Laparoscopic
Kalata et al., [3]	USA	Retrospective Cohort	Total: 1,026,088 LC: 1,001,004 RAC: 25,084	LC: 72 ± 12 years;RAC: 71.2 ± 11.6 years	LC: 467,612/ 1,001,004 (46.7%) RAC: 11,424/ 25,084 (45.5%)	Robotic-Assisted, Laparoscopic
Bedeir et al., [7]	USA	Retrospective cohort study	Total: 458 LC: 281 RAC: 177	N/A	N/A	Laparoscopic Cholecystectomy versus Robotic Single-Site Cholecystectomy (RSSC)
Li et al., [10]	Taiwan	Retrospective cohort study	Total: 445 LC: 367 RC: 78	LC: mean 51.44 ± 14.11; RAC: mean 59.69 ± 13.35	CLC: 161/367 (43.9%); RC: 37/78 (48.3%)	Conventional laparoscopic cholecystectomy (CLC) vs single-site robotic cholecystectomy (RC)
Rifai et al., [11]	USA	Retrospective cohort study	Total: 270 LC: 105 RAC: 165	LC: mean 45.90 years (range 19–86); RAC: mean 51.98 years (range 19–101)	LC: 36/105 males (34.3%); RAC: 67/165 males (40.6%)	Standard Laparoscopic vs. Robotic-Assisted approach
Grochola et al., [12]	Switzerland	Single-centre, single-blinded randomized controlled trial	Total: 60 LC: 30 RAC: 30	SILC: mean 51.5 years (range 30–78); dVSSC: mean 52.4 years (range 26–82)	LC: 14/30 (46.7%); dVSSC: 10/30 (33.3%)	Single-incision laparoscopic cholecystectomy (SILC) vs da Vinci Single-Site cholecystectomy (dVSSC)

All data extracted were tabulated and recorded on a Microsoft Excel sheet.

### Quality assessment

The Newcastle- Ottawa Scale (NOS) was used to assess the quality of included studies identified from our search protocol [9] (Table 2). The NOS assesses studies in three domains, including the selection of groups, comparability between groups, and the outcomes of cohort studies. It utilizes a point-based system and each study is given a point between 0 to 9. Studies with scores of 7 or greater were considered as high quality.

**Table 2:** Outcomes from assessment of risk bias of cohort studies using the Newcastle–Ottawa Scale [9]

First author, year of publication	Total quality score (Max 9)	Selection (Max 4)	Comparability (Max 2)	Exposure/Outcome (Max 3)
Pokala et al., [2]	7	****	*	**
Kalata et al., [3]	8	****	**	**
Bedeir et al., [7]	6	***	*	**
Li et al., [10]	6	***	*	**
Rifai et al., [11]	6	***	*	**

Review authors worked independently in assessing the quality of studies and results were compared. Any differences in scores was resolved via a thorough discussion between the authors.

### Data analysis

Continuous variables were presented as the mean and standard deviation (SD), while categorical variables were expressed as percentages.

A meta-analysis was conducted to determine the comparative effectiveness of LC and RAC. A meta-analysis for operative time was planned; however, pooling was not performed because only one study reported data in a format suitable for quantitative synthesis. Since substantial heterogeneity among the studies was expected, a random-effects model was applied. The I^2^ statistic was used to determine the magnitude of variability between included studies, with the value of more than 75% denoting considerable heterogeneity. A Z-test was also conducted to estimate the overall effect with the level of statistical significance at P < 0.05.

All statistical analyses were carried out using Review Manager (RevMan) [Computer program], Version 5.4, The Cochrane Collaboration, 2020.

## Results

### Study selection

The searches identified a total of 228 studies, 169 of which were found to be duplicates and therefore removed before continuing to the next step. Titles and abstracts of the remaining 59 studies were screened, leading to the exclusion of 51 studies. We then requested the full texts of the 8 remaining studies and assessed them against the set inclusion and exclusion criteria.

Two studies was identified that initially met inclusion and exclusion criteria but were then excluded for the following reasons:

- Kossenas et al.,[6]: Findings were narrowly focused on a BMI-specific patient population, which limited the findings’ broader applicability in general gallbladder surgical cases [6].
- Singh et al., [4]: Excluded because it was a model-based cost-effectiveness analysis using secondary/published data, not a primary clinical comparative study [4].

**Fig. 1:**
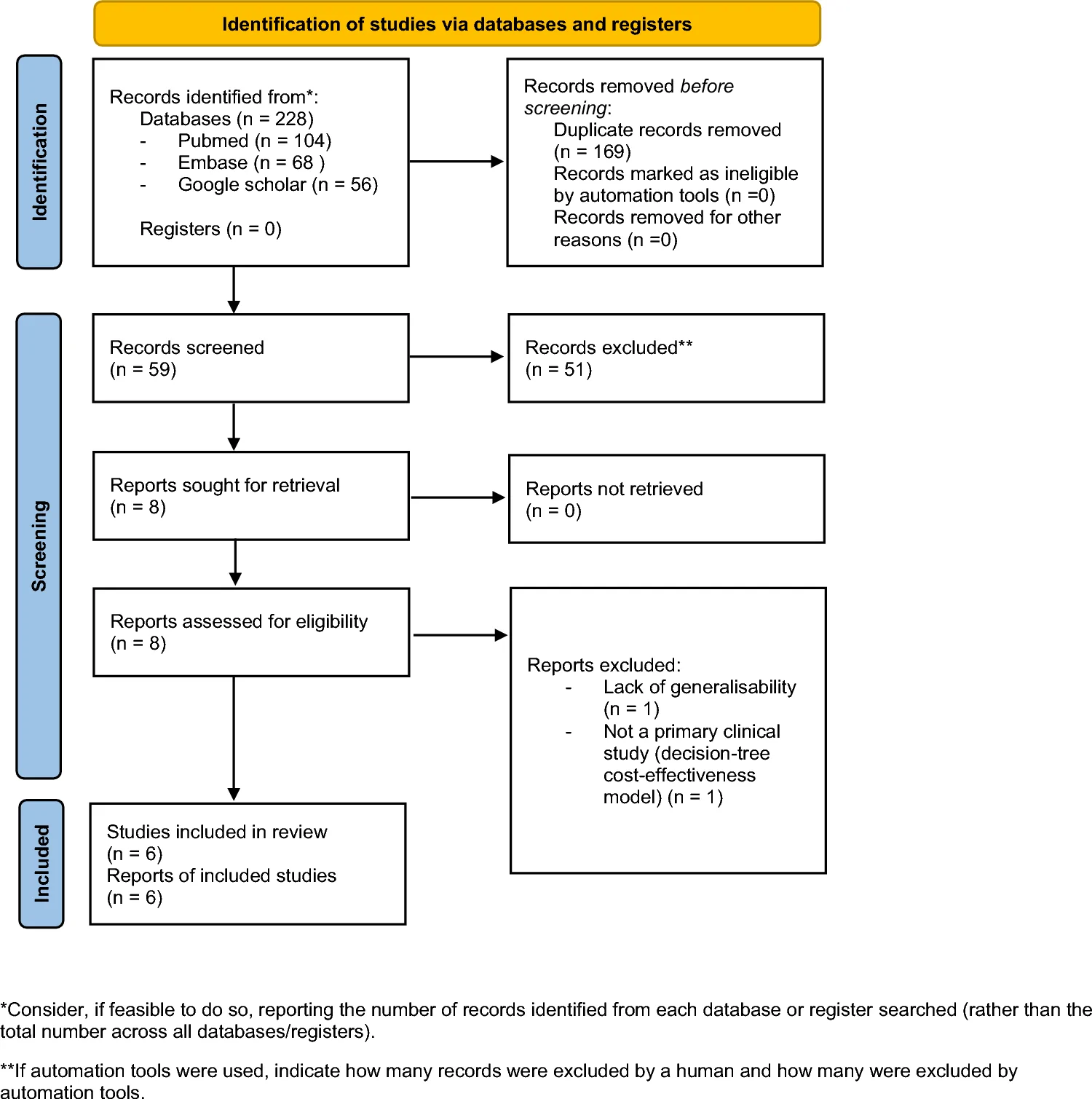
Preferred Reporting Items for Systematic Reviews and Meta-analyses flow diagram, 2020 version for searches of databases and registers only [8]

Finally, 6 studies were found suitable for data extraction and analysis (Table 3).PRISMA 2020 flow diagram for new systematic reviews which included searches of databases and registers only (Fig. 1).

**Table 3:** Results of individual studies

Author (s), Year of publication	Operatlive time (min)	Complication rates (n) %	Post op length of hospital stay (days) mean ± SD	Conversion rates (n) %	Postoperative pain	Cost-Effectiveness
Pokala et al. [2]	N/A	LC: 851/89,878 (0.9%); RC: 34/1,971 (1.7%)	LC: 3.10 ± 2.22; RC: 3.27 ± 2.72	N/A	No direct pain score reported; opiate prescription/utilization used as a surrogate. LC: 98.3% prescribed opiates; RC: 97.2%	RC was not cost-effective / more costly with no added benefit. Total cost: LC: $6,503 ± 3,706; RC: $8,620 ± 5,055
Kalata et al., [3]	N/A	Overall 30-day complications: RAC 20.5% vs LC 20.6%; Serious complications: RAC 9.3% vs LC 8.6%; Bile duct injury requiring repair: RAC 0.7% vs LC 0.2%	N/A	N/A	N/A	N/A
Bedeir et al., [7]	early RSSC 98 min (IQR 92–114) vs late RSSC 87 min (IQR 75–95)	RSSC: no common/hepatic duct injuries and no bile leak reported; overall comparative complication rate vs LC not reported	N/A	RSSC: 0/177 converted to LC or open (0%); LC conversion rate not reported	N/A	In elective outpatient cost analysis, RSSC was cheaper than LC: median total cost $1319 vs $1737
Li et al., [10]	CLC: 64.37 ± 30.61 min vs RC: 75.7 ± 31.3 min (*p*= 0.035)	CLC: 75/367 (20.4%); RC: 3/78 (3.8%)	CLC: 3.73 ± 1.77; RC: 4.35 ± 0.75	CLC: 7/367 (1.9%); RC: 0/78 (0%)	No direct pain score reported. Analgesics requirement duration: CLC: 1.13 ± 3.30 days; RC: 0.64 ± 2.11 days	Not cost-effective for RC in index hospitalization. Hospital charge: CLC: NTD 49,218.01 ± 15,324.59; RC: NTD 204,125 ± 47,037.03
Rifai et al., [11]	LC: 101.52 (Mean); RC: 98.04 (Mean)	N/A	N/A	LC: 9/105 (8.6%); RAC: 0/165 (0%)	N/A	N/A
Grochola et al., [12]	SILC: 74 min (31–135) vs dVSSC: 85.5 min (48–148); also console time (dVSSC): 35.0 min (21–107)	Any 30-day complication: SILC: 7/30 (23.3%); dVSSC: 4/30 (13.3%)	SILC: mean 3.06 days (range 1–26); dVSSC: mean 1.9 days (range 1–4)	Conversion to conventional laparoscopy: SILC: 3/30 (10.0%); dVSSC: 2/30 (6.7%); Conversion to open: 0/30 in both groups	No direct postoperative pain score reported in this trial. The discussion mentions prior SILC evidence on pain, but this study itself assessed surgeon stress load, HRQoL, and cosmesis rather than postoperative pain	SILC: $8,284; dVSSC: $12,219 RC costs significantly higher

### Study characteristics

#### Assessments of risk of bias in studies

The methodological quality of the included cohort studies was assessed using the Newcastle–Ottawa Scale. Overall, study quality ranged from moderate to high, with total NOS scores between 6 and 8. Kalata et al. achieved the highest score because of its large population-based design and stronger adjustment for confounding, whereas the remaining retrospective single-center cohort studies were judged to be of moderate quality.

### Results of individual studies

#### Primary outcome

**Fig. 2:**
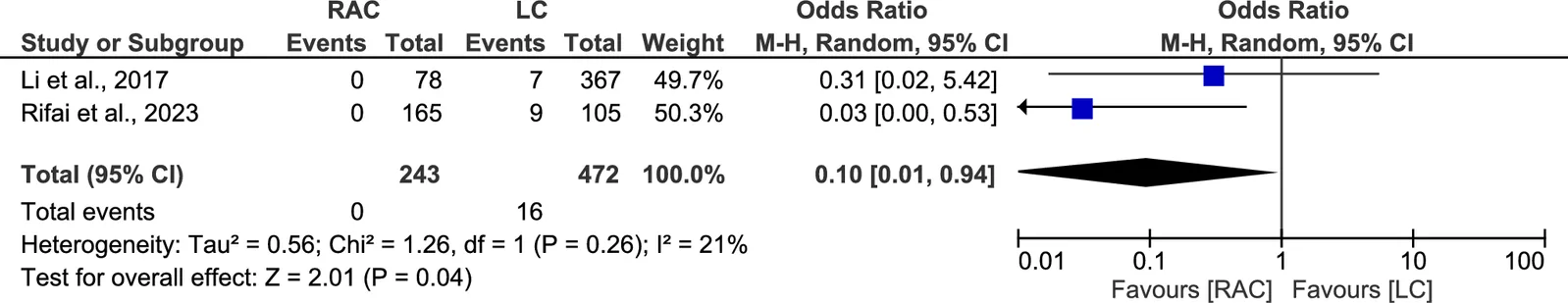
Forest plot illustrating the odds ratios for conversion rates to open surgery between RAC and LC

Meta-analysis of conversion to open surgery demonstrated that RAC was associated with significantly lower odds of conversion than LC (OR 0.10, 95% CI 0.01–0.94;* p* = 0.04) (Fig. 2). This analysis included two retrospective comparative studies. In Li et al. [10], no conversions occurred in the RAC group (0/78) compared with 7/367 in the LC group [10]. Rifai et al. [11] similarly reported 0/165 conversions in the RAC group versus 9/105 in the LC group [11]. Statistical heterogeneity was low (I^2^ = 21%). Despite the pooled effect favoring RAC, this finding should be interpreted cautiously given the limited number of included studies, retrospective study designs, and sparse-event structure of the data.

### Secondary outcomes

#### Operative time

Operative time was not meta-analyzed because only one study provided directly usable continuous data (mean ± SD) for both RAC and LC. In that study, Li et al. [10] reported a significantly longer operative time in the RAC group compared with the LC group (75.7 ± 31.3 min vs 64.37 ± 30.61 min; *p*= 0.035) [10]. Another study, Rifai et al. [11], reported mean operative times for cholecystectomy (1.634 h for RAC vs 1.692 h for LC) but did not provide standard deviations, precluding quantitative pooling [11]. Therefore, operative time was summarized narratively.

#### Post-op length of hospital stay

**Fig. 3:**
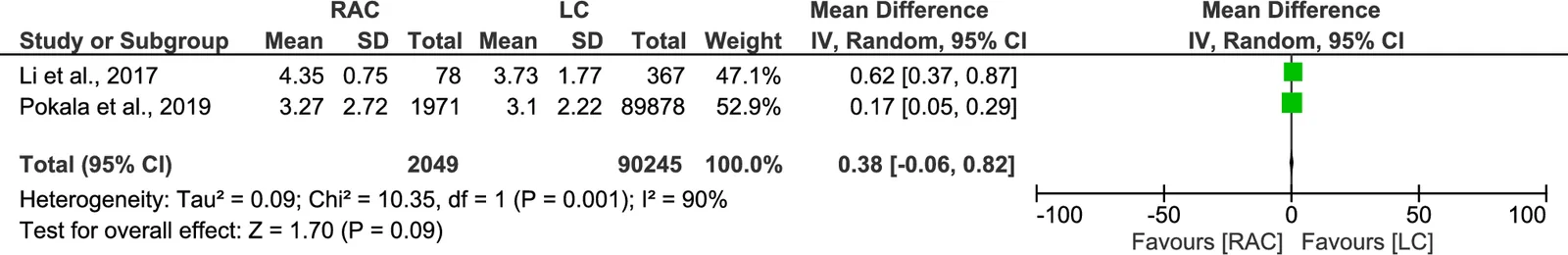
Forest plot illustrating the mean difference in post-operative length of hospital stay (in days) between RAC and LC

Meta-analysis of post-operative length of hospital stay included two studies with directly extractable mean ± SD data and showed no statistically significant difference between RAC and LC (MD 0.38 days, 95% CI -0.06 to 0.82;* p*=  0.09), although heterogeneity was substantial (I^2^ = 90%) (Fig. 3). In both included studies, hospital stay was numerically longer in the RAC group than in the LC group. Li et al. [10] reported a mean stay of 4.35 ± 0.75 days in the RAC group versus 3.73 ± 1.77 days in the LC group [10], while Pokala et al. [2] reported 3.27 ± 2.72 days versus 3.10 ± 2.22 days, respectively [2]. Overall, although both studies numerically favored LC, the pooled estimate did not demonstrate a significant difference between approaches. The high heterogeneity suggests that differences in study design, patient selection, and clinical setting may have influenced the observed effect.

Additionally, as Grochola et al. reported length of stay as mean with median and range, but without standard deviation, the study was excluded from the primary meta-analysis; estimation of SD from the reported range was considered exploratory only because the data appeared skewed [12].

#### Complication rates

**Fig. 4:**
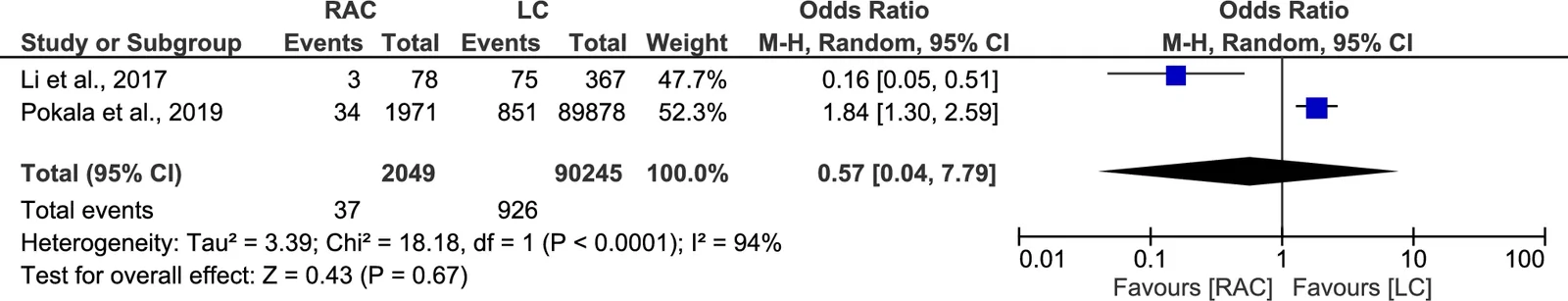
Forest plot of overall complication rates comparing RAC and LC expressed as odds ratios

Meta-analysis of postoperative complication rates included two studies and showed no statistically significant difference between RAC and LC overall (OR 0.57, 95% CI 0.04–7.79;* p* = 0.67) (Fig. 4). In Li et al. [10], postoperative complications occurred in 3/78 (3.8%) patients in the RAC group and 75/367 (20.4%) in the LC group, favoring RAC [10]. In contrast, Pokala et al. [2] reported complications in 34/1,971 (1.7%) RAC cases and 851/89,878 (0.9%) LC cases, favoring LC [2]. There was considerable heterogeneity between studies (I^2^ = 94%), indicating substantial inconsistency in the direction and magnitude of effect estimates. Accordingly, although the pooled analysis did not demonstrate a significant difference in postoperative complication rates between approaches, this result should be interpreted cautiously.

#### Post-operative pain

Post-operative pain was not assessed using standardized methods across the included studies, and none provided a clearly described standardized analgesia protocol for direct comparison. Li et al. [10] did not use a formal pain scale, but reported a shorter duration of analgesic requirement in the robotic group than in the laparoscopic group (0.64 ± 2.11 vs 1.13 ± 3.30 days; *p* = 0.023) [10]. Pokala et al. [2] similarly did not report pain scores, instead evaluating in-hospital opiate utilization from an administrative database and finding a slightly lower prescription frequency in the robotic group (97.2% vs 98.3%) [2]. Because pain was measured indirectly and analgesia protocols were not specified, these findings should be interpreted with caution. Any apparent difference in post-operative pain may reflect variation in prescribing practice, perioperative care pathways, or case selection rather than a direct effect of the robotic platform itself.

#### Cost-effectiveness

Cost and cost-effectiveness were summarized narratively because economic outcomes were reported heterogeneously across studies, with differences in currency, costing methodology, and study design. Overall, most included studies suggested that robotic cholecystectomy was associated with higher cost than laparoscopic cholecystectomy. Pokala et al. reported higher total cost for robotic-assisted cholecystectomy than laparoscopic cholecystectomy ($8620 ± 5,55 vs $6503 ± 3,06) [2]. Similarly, Li et al. found substantially higher hospital charges in the robotic group than in the conventional laparoscopic group [10]. In contrast, Bedeir et al., in a restricted cohort of elective outpatient cases at a center with pre-existing robotic infrastructure, reported lower median total procedural cost for robotic single-site cholecystectomy compared with laparoscopic cholecystectomy ($1319 vs $1737) [7].

## Discussion

This review suggests that RAC may offer a technical advantage in selected cases, but the current evidence does not demonstrate consistent overall superiority over LC. Although the signal toward lower conversion with RAC is noteworthy, the broader pattern across secondary outcomes remains mixed, and economic findings generally favor LC.

The lower conversion signal observed with RAC may reflect advantages of the robotic platform in dissection and instrument control. However, this possible benefit was not accompanied by a clear and consistent advantage across other perioperative outcomes. The absence of a reliable reduction in hospital stay or complications suggests that any technical strengths of RAC may be offset by differences in case selection, perioperative management, and institutional workflow.

Safety interpretation also remains cautious. The pooled complication analysis did not identify a significant difference, but the direction of effect varied between studies and heterogeneity was substantial. This suggests that complication profiles may be influenced by differences in study design, outcome definitions, and clinical setting rather than by surgical platform alone. Accordingly, the present evidence does not support strong claims of a safety advantage for either approach.

Operative efficiency is similarly difficult to interpret. Operative time was reported inconsistently across the included studies, and this limits confidence in broader conclusions. In practice, operative duration is likely influenced not only by the platform itself, but also by surgeon experience, team familiarity, setup and docking processes, and local theatre workflow. From a service-delivery perspective, this is relevant because prolonged operating-room utilization may have implications beyond the individual patient, including theatre efficiency, resource allocation, and potentially waiting-list capacity. This point is particularly important when considering a high-volume procedure such as cholecystectomy.

Postoperative pain should also be interpreted with caution. The included studies did not report clearly standardized analgesia protocols, and pain outcomes were not assessed using uniform validated measures. Li et al. used duration of analgesic requirement as a surrogate rather than a formal pain score, while Pokala et al. reported in-hospital opiate prescribing and utilization from administrative data rather than direct patient-reported pain outcomes [2]. As a result, any apparent difference in pain-related outcomes cannot be confidently attributed to the robotic platform itself and may instead reflect variation in prescribing practice, perioperative care pathways, or case mix. For this reason, it would be overly simplistic to interpret reduced analgesic burden as a direct consequence of greater robotic precision.

Economic findings were more consistent in direction than clinical outcomes. Most studies suggested higher cost with RAC, which is in keeping with the increased supply and operating-room costs reported in larger datasets. At the same time, the lower procedural costs reported in selected outpatient robotic single-site practice by Bedeir et al. indicate that local infrastructure, case selection, and institutional experience may substantially influence economic performance. Cost conclusions should therefore be interpreted within the context of the health system and operative model in which RAC is implemented.

A strength of this review is that it considered both clinical and economic outcomes and applied quantitative synthesis where direct pooling was methodologically appropriate. Several limitations should also be acknowledged. Most included studies were retrospective and therefore prone to selection bias and residual confounding. Only a small number of studies contributed to each pooled analysis, limiting precision. Several outcomes were incompletely reported or measured using non-comparable methods, restricting quantitative synthesis. In addition, the included studies were not uniform with respect to robotic technique, as some evaluated single-site robotic cholecystectomy whereas others assessed robotic-assisted cholecystectomy more broadly; this variation may have contributed to heterogeneity across outcomes. Finally, economic findings may not be fully generalizable across institutions with different volumes, reimbursement models, and access to robotic infrastructure.

Taken together, the present evidence does not support a clear overall perioperative advantage of RAC over LC for benign gallbladder disease. RAC may have a role in selected settings, particularly where surgeon expertise and institutional support are established, but any potential technical benefit must be balanced against uncertain broader clinical gains and greater economic burden. Future comparative studies should use standardized perioperative outcome definitions, clearly report analgesia protocols, distinguish between robotic techniques, and include more rigorous evaluation of theatre utilization and cost sustainability.

Overall, RAC may reduce conversion in selected settings, but broader clinical benefit remains unproven. Given the lack of consistent advantage across secondary outcomes and the generally higher cost of robotic surgery, LC remains the more established and economically justifiable approach for most patients undergoing cholecystectomy for benign gallbladder disease.

## Appendix

**Table:**

Section and topic	Item	Checklist item	
*Title*			
Title	1	Identify the report as a systematic review	
*Abstract*			
Abstract	2	See the PRISMA 2020 for Abstracts checklist.	
*Introduction*			
Rationale	3	Describe the rationale for the review in the context of existing knowledge.	
Objectives	4	Provide an explicit statement of the objective(s) or question(s) the review addresses.	
*Methods*			
Eligibility criteria	5	Specify the inclusion and exclusion criteria for the review and how studies were grouped for the syntheses.	
Information sources	6	Specify all databases, registers, websites, organisations, reference lists and other sources searched or consulted to identify studies. Specify the date when each source was last searched or consulted.	
Search strategy	7	Present the full search strategies for all databases, registers and websites, including any filters and limits used.	
Selection process	8	Specify the methods used to decide whether a study met the inclusion criteria of the review, including how many reviewers screened each record and each report retrieved, whether they worked independently, and if applicable, details of automation tools used in the process.	
Data collection process	9	Specify the methods used to collect data from reports, including how many reviewers collected data from each report, whether they worked independently, any processes for obtaining or confirming data from study investigators, and if applicable, details of automation tools used in the process.	
Data items	10a	List and define all outcomes for which data were sought. Specify whether all results that were compatible with each outcome domain in each study were sought (e.g. for all measures, time points, analyses), and if not, the methods used to decide which results to collect.	
	10b	List and define all other variables for which data were sought (e.g. participant and intervention characteristics, funding sources). Describe any assumptions made about any missing or unclear information.	
Study risk of bias assessment	11	Specify the methods used to assess risk of bias in the included studies, including details of the tool(s) used, how many reviewers assessed each study and whether they worked independently, and if applicable, details of automation tools used in the process.	
Effect measures	12	Specify for each outcome the effect measure(s) (e.g. risk ratio, mean difference) used in the synthesis or presentation of results.	
Synthesis methods	13a	Describe the processes used to decide which studies were eligible for each synthesis (e.g. tabulating the study intervention characteristics and comparing against the planned groups for each synthesis (item #5)).	
	13b	Describe any methods required to prepare the data for presentation or synthesis, such as handling of missing summary statistics, or data conversions.	
	13c	Describe any methods used to tabulate or visually display results of individual studies and syntheses.Describe any methods used to tabulate or visually display results of individual studies and syntheses.	
	13d	Describe any methods used to synthesize results and provide a rationale for the choice(s). If meta-analysis was performed, describe the model(s), method(s) to identify the presence and extent of statistical heterogeneity, and software package(s) used.	
	13e	Describe any methods used to explore possible causes of heterogeneity among study results (e.g. subgroup analysis, meta-regression).	
	13f	Describe any sensitivity analyses conducted to assess robustness of the synthesized results.	
Reporting bias assessment	14	Describe any methods used to assess risk of bias due to missing results in a synthesis (arising from reporting biases).	
Certainty assessment	15	Describe any methods used to assess certainty (or confidence) in the body of evidence for an outcome.	
*Results*			
Study selection	16a	Describe the resul ts of the search and selection process, from the number of records identified in the search to the number of studies included in the review, ideally using a flow diagram.	
	16b	Cite studies that might appear to meet the inclusion criteria, but which were excluded, and explain why they were excluded.	
Study characteristics	17	Cite each included study and present its characteristics.	
Risk of bias in studies	18	Present assessments of risk of bias for each included study.	
Results of individual studies	19	For all outcomes, present, for each study: (a) summary statistics for each group (where appropriate) and (b) an effect estimate and its precision (e.g. confidence/credible interval), ideally using structured tables or plots.	
Results of syntheses	20a	For each synthesis, briefly summarise the characteristics and risk of bias among contributing studies.	
	20b	Present results of all statistical syntheses conducted. If meta-analysis was done, present for each the summary estimate and its precision (e.g. confidence/credible interval) and measures of statistical heterogeneity. If comparing groups, describe the direction of the effect.	
	20c	Present results of all investigations of possible causes of heterogeneity among study results.	
	20d	Present results of all sensitivity analyses conducted to assess the robustness of the synthesized results.	
Reporting biases	21	Present assessments of risk of bias due to missing results (arising from reporting biases) for each synthesis assessed.	
Certainty of evidence	22	Present assessments of certainty (or confidence) in the body of evidence for each outcome assessed.	
*Discussion*			
Discussion	23a	Provide a general interpretation of the results in the context of other evidence.	
	23b	Discuss any limitations of the evidence included in the review.	
	23c	Discuss any limitations of the review processes used.	
	23d	Discuss implications of the results for practice, policy, and future research.	
*Other information*			
Registration and protocol	24a	Provide registration information for the review, including register name and registration number, or state that the review was not registered.	
	24b	Indicate where the review protocol can be accessed, or state that a protocol was not prepared.	
	24c	Describe and explain any amendments to information provided at registration or in the protocol.	
Support	25	Describe sources of financial or non-financial support for the review, and the role of the funders or sponsors in the review.	
Competing interests	26	Declare any competing interests of review authors.	
Availability of data, code and other materials	27	Report which of the following are publicly available and where they can be found: template data collection forms; data extracted from included studies; data used for all analyses; analytic code; any other materials used in the review.	

From: Page MJ, McKenzie JE, Bossuyt PM, Boutron I, Hoffmann TC, Mulrow CD, et al. The PRISMA 2020 statement: an updated guideline for reporting systematic reviews. BMJ 2021;372:n71. doi: 10.1136/bmj.n71. This work is licensed under CC BY 4.0. To view a copy of this license, visit https://creativecommons.org/licenses/by/4.0/

## Abbreviations

LC: Laparoscopic cholecystectomy
RAC: Robotic-assisted cholecystectomy
CLC: Conventional laparoscopic cholecystectomy
RSSC: Robotic single-site cholecystectomy
SILC: Single-incision laparoscopic cholecystectomy
dVSSC: Da Vinci Single-Site cholecystectomy
NOS: Newcastle- Ottawa Scale
PRISMA: Preferred reporting items for systematic reviews and meta- analysis
OR: Odds ratio
CI: Confidence interval
SD: Standard deviation
I^2^: Heterogeneity statistic
IQR: Interquartile range
ICER: Incremental cost-effectiveness ratio
QALY: Quality-adjusted life-year
MD: Mean difference
